# Editorial: Bridging Inflammation, Oxidative Stress, and Metabolic Dysfunction in Brain Disorders

**DOI:** 10.3390/brainsci15111211

**Published:** 2025-11-10

**Authors:** Ansab Akhtar

**Affiliations:** Neuroscience Center, School of Medicine, LSU Health, New Orleans, LA 70112, USA; ansabakhtar@gmail.com

## 1. Introduction: The Interconnected Landscape of Brain Pathophysiology

We present this Special Issue of *Brain Sciences*, titled “Role of Inflammation, Oxidative Stress, and Metabolic Dysfunction in Neurodevelopmental and Neurodegenerative Diseases: Pharmacological Targets and Therapeutic Interventions”, which brings together research and review articles that explore the molecular crosstalk between neuroinflammatory, oxidative stress, and metabolic pathways underlying both neurodegenerative and neurodevelopmental disorders. The collection represents an important step toward understanding how cellular energy metabolism, redox homeostasis, and inflammatory signaling converge to determine brain health and disease outcomes.

Over the past two decades, neuroscience has increasingly recognized that neuroinflammation, oxidative imbalance, and metabolic dysfunction are not isolated processes but intricately linked biological phenomena. For example, oxidative stress can initiate inflammatory cascades through the activation of nuclear factor kappa B (NF-κB) and other transcriptional regulators [1]. At the same time, inflammation exacerbates oxidative stress via reactive oxygen and nitrogen species (ROS/RNS) production. Mitochondrial dysfunction lies at the intersection of these processes, impairing cellular energy metabolism and promoting neuronal vulnerability [1,2]. These interconnected mechanisms contribute to diseases such as Alzheimer’s disease (AD), Parkinson’s disease (PD), autism spectrum disorder (ASD), and diabetes-related cognitive decline.

Research has also revealed that metabolic signals can act as immunomodulators, where adenosine triphosphate (ATP), NAD+, and lipid mediators influence the balance between neuroprotective and neurotoxic glial states. The emerging field of immunometabolism underscores that inflammation and metabolism share common molecular regulators such as AMP-activated protein kinase (AMPK), peroxisome proliferator-activated receptors (PPARs), and sirtuins [3,4]. This Special Issue, therefore, provides a platform for studies examining these overlapping processes at the molecular, behavioral, and therapeutic levels.

## 2. Overview of This Special Issue’s Themes

The contributions in this Special Issue span preclinical, computational, and integrative approaches addressing brain disorders linked to inflammation, oxidative stress, and metabolism. Collectively, they can be organized under four broad themes:

- Neuroprotection through pharmacological and natural compounds.
- Mitochondrial dysfunction and metabolic stress in neurodevelopmental disorders.
- Neurodegenerative models linking oxidative and inflammatory mechanisms.
- Crosstalk between metabolic regulators and neuroimmune signaling.

Each article enriches our understanding of how neuroinflammation and redox imbalance may be therapeutically modulated through pharmacological, nutraceutical, and computational strategies.

## 3. Details About This Special Issue’s Contributions 

The contributions in this Special Issue highlight diverse therapeutic strategies that target multiple aspects of the oxidative–inflammatory–metabolic triad.

### 3.1. Motor Function Improvement During Early Development

This research article “Vinpocetine, a Phosphodiesterase Type 1 Inhibitor, Mitigates Locomotor Hyperactivity in Female Mice Exposed to Lead During Development” (contribution 1), investigates whether vinpocetine, a phosphodiesterase-1 (PDE1) inhibitor, can reverse hyperactive locomotor behavior in female mice exposed to developmental lead toxicity. Using a rodent model where lead exposure occurred during gestation and early postnatal periods, the authors found that lead exposure selectively heightened locomotion in females and that administration of vinpocetine significantly attenuated this hyperactivity. The study suggests that disruptions in cAMP/cGMP signaling contribute to lead-induced behavioral abnormalities, and that modulation of cyclic nucleotide pathways may offer therapeutic potential. The findings also highlight sex-dependent vulnerability in neurobehavioral toxicity and support further exploration of this PDE1 inhibitor in models of developmental neurotoxicity and behavioral disorders.

### 3.2. Plant-Derived Compounds and Phytochemicals in the Depression Model

The paper “*Eugenia uniflora* Effects on the Depressive-like Behavior of MPTP-Exposed Female Rats: Apoptosis and α-Synuclein Modulation” investigates how *Eugenia uniflora* (contribution 2) extract mitigates depressive-like behavior and neurodegeneration by regulating apoptotic pathways and reducing α-synuclein aggregation. The findings suggest that flavonoid- and phenolic-rich plant extracts can simultaneously attenuate oxidative damage and restore neurochemical balance, presenting a viable complementary strategy for Parkinson’s disease.

Essentially, this experimental study demonstrates that *Eugenia uniflora* extract mitigates depressive-like behaviors and neurodegeneration in MPTP-induced rats by reducing apoptosis and α-synuclein accumulation, highlighting plant-derived antioxidants as potential neuroprotective agents.

### 3.3. Repurposed Drugs and Metabolic Regulation

Another report, “In Vivo and Computational Studies on Sitagliptin’s Neuroprotective Role in Type 2 Diabetes Mellitus: Implications for Alzheimer’s Disease” (contribution 3), explores how an anti-diabetic drug can exert neuroprotective effects. Sitagliptin, by modulating DPP-4 and enhancing incretin signaling, reduces oxidative and inflammatory stress in neuronal tissue. Computational modeling revealed strong docking interactions with Alzheimer’s-associated proteins, indicating crosstalk between glucose regulation and neuroprotection [5].

Here, the authors combine biochemical, behavioral, and molecular docking analyses to show how sitagliptin, a DPP-4 inhibitor, attenuates oxidative damage, normalizes neurotransmitter levels, and protects neurons from diabetes-induced metabolic stress.

### 3.4. Mitochondrial Dysfunction and Neurodevelopmental Disorders

A review paper, “The Interplay of Oxidative Stress, Mitochondrial Dysfunction, and Neuroinflammation in Autism Spectrum Disorder: Behavioral Implications and Therapeutic Strategies” (contribution 4), comprehensively reviews how early-life oxidative and inflammatory insults can impair neuronal connectivity and synaptic signaling. Mitochondrial impairment in ASD leads to reduced ATP production and altered calcium buffering, which contribute to behavioral abnormalities. The review also discusses potential interventions such as flavonoids, coenzyme Q10, omega-3 fatty acids, etc.

This literature review provides compelling evidence that oxidative stress and mitochondrial dysfunction are not only features of neurodegeneration but also play a causal role in atypical neurodevelopment. By linking cellular metabolism to behavior, the paper bridges basic neuroscience with clinical relevance.

Hence, this comprehensive review integrates findings on mitochondrial impairment and chronic neuroinflammation in ASD. It discusses therapeutic interventions, from antioxidants and metabolic regulators to behavioral therapies, aimed at restoring redox equilibrium.

### 3.5. Cellular Signaling and Amyloid Clearance

Another review, “Clearing Amyloid-Beta by Astrocytes: The Role of Rho GTPases Signaling Pathways as Potential Therapeutic Targets” (contribution 5), focuses on astrocytic regulation of amyloid-β clearance. It suggests that targeting Rho GTPase signaling could enhance cytoskeletal dynamics and promote lysosomal degradation of amyloid plaques. The study deepens our understanding of glial mechanisms and opens new therapeutic possibilities beyond traditional neuron-centered approaches.

This is a detailed review emphasizing astrocyte-mediated clearance of amyloid-β via Rho GTPase signaling. The authors propose targeting cytoskeletal dynamics as a promising therapeutic strategy in Alzheimer’s pathology [5].

### 3.6. Neutrophic Factor and Antioxidant-Mediated Neuronal Defense

In the review “Indole-3-Carbinol and Its Derivatives as Neuroprotective Modulators” (contribution 6), the authors explore how indole-3-carbinol and related phytochemicals act as multi-target agents capable of modulating oxidative stress, inflammatory mediators, and apoptosis-related signaling. 

The overview provides mechanistic insights into how indole-3-carbinol activates antioxidant defenses via the Nrf2/ARE pathway and modulates inflammatory cytokine release. The review underscores that nutraceutical compounds capable of acting on multiple molecular targets are valuable tools in preventive neurotherapeutics. 

Together, these papers offer a multidimensional overview of how convergent biological processes shape brain health. By bridging pharmacological, nutritional, and molecular approaches, this Special Issue illustrates that targeting shared pathophysiological mechanisms may yield broader therapeutic benefits than addressing isolated pathways.

## 4. Integrative Insights, Limitations, and Emerging Directions

The articles collectively point toward three emerging paradigms in neurotherapeutics:

*Systems Integration*: Multi-omics and computational modeling are enabling researchers to view brain disorders as network-level phenomena rather than single-pathway dysfunctions.

*Temporal Sensitivity*: The timing and intensity of inflammatory and redox processes determine whether they are adaptive or pathological. Understanding these dynamics is crucial for designing stage-specific interventions. 

*Metabolic Resilience*: Enhancing mitochondrial efficiency and redox balance represents a cornerstone strategy in neuroprotection [6].

Recent evidence indicates that the gut–brain axis also plays a role in linking systemic metabolism with neuroinflammation, mediated by microbiota-derived metabolites such as short-chain fatty acids [7,8]. The cross-disciplinary implications are profound, suggesting that interventions in diet, metabolism, and immunity can converge to promote cognitive health [9].

Despite these advances, several gaps remain. Translating preclinical findings into human applications is hindered by biological complexity, species differences, and methodological variability. Many antioxidant trials have yielded inconsistent results due to poor bioavailability and lack of target specificity. Furthermore, the chronicity of human neurodegenerative diseases often involves feedback loops that simple antioxidant therapies cannot interrupt.

Emerging research must therefore focus on multi-target therapies and network pharmacology, integrating insights from systems biology to develop interventions that simultaneously modulate multiple molecular pathways. Another critical frontier is biomarker development. Quantitative imaging of oxidative stress, plasma metabolite profiling, and inflammatory cytokine panels can improve diagnostic precision and monitor treatment response [10].

International collaboration and open-data initiatives will be essential for establishing reproducible models and standardized endpoints. Integrating genomic, proteomic, and metabolomic data could further advance the development of predictive models that capture the true complexity of neurological disorders.

## 5. Conclusions and Future Perspectives

This Special Issue underscores the vital importance of integrating metabolic, oxidative, and inflammatory research in neuroscience. The brain’s resilience depends on maintaining harmony among these systems. The studies presented here demonstrate that effective therapeutic strategies must be multifaceted, targeting not only the symptoms but also the cellular stress networks that drive neurodegeneration and developmental pathology.

Moving forward, collaboration between molecular neuroscientists, pharmacologists, and clinicians will be key. As the field moves toward personalized medicine, biomarkers derived from oxidative and metabolic signatures will help tailor preventive and therapeutic interventions. Ultimately, this integrated approach promises not only improved treatment outcomes but also a deeper understanding of the brain’s adaptive complexity.
